# A professional knowledge base for collaborative reflection education: a qualitative description of teacher goals and strategies

**DOI:** 10.1007/s40037-021-00677-6

**Published:** 2021-08-17

**Authors:** Marije van Braak, Mario Veen, Jean Muris, Pieter van den Berg, Esther Giroldi

**Affiliations:** 1 Department of General Practice, Erasmus Medical Center, Rotterdam, The Netherlands; 2 Department of Family Medicine, Maastricht University, Care and Public Health Research Institute (CAPHRI), Maastricht, The Netherlands; 3 Department of Educational Development and Research, Maastricht University, School of Health Professions Education (SHE), Maastricht, The Netherlands

**Keywords:** Collaborative reflection, Professional knowledge base, Professional Stocks of Interactional Knowledge, Teacher professional development

## Abstract

**Introduction:**

For several decades, educational experts have promoted reflection as essential to professional development. In the medical setting, collaborative reflection has gained significant importance across the curriculum. Collaborative reflection has a unique edge over individual reflection, but many medical teachers find facilitating group reflection sessions challenging and there is little documentation about the didactics of teaching in such collaborative reflection settings. To address this knowledge gap, we aim to capture the professional knowledge base for facilitating collaborative reflection by analyzing the formal and perceived goals and strategies of this practice.

**Methods:**

The professional knowledge base consists of formal curricular materials as well as individual teacher expertise. Using Template Analysis, we analyzed the goals and strategies of collaborative reflection reported in institutional training documents and video-stimulated interviews with individual teachers across all Dutch general practitioner training institutes.

**Results:**

The analysis resulted in a highly diverse overview of educational goals for residents during the sessions, teacher goals that contribute to those educational goals, and a myriad of situation-specific teacher strategies to accomplish both types of goals. Teachers reported that the main educational goal was for residents to learn and develop and that the teachers’ main goal was to facilitate learning and development by ensuring everyone’s participation in reflection. Key teacher strategies to that end were to manage participation, to ensure a safe learning environment, and to create conditions for learning.

**Discussion:**

The variety of strategies and goals that constitute the professional knowledge base for facilitating collaborative reflection in postgraduate medical education shows how diverse and situation-dependent such facilitation can be. Our analysis identifies a repertoire of tools that both novice and experienced teachers can use to develop their professional skill in facilitating collaborative reflection.

**Supplementary Information:**

The online version of this article (10.1007/s40037-021-00677-6) contains supplementary material, which is available to authorized users.

## Introduction

Collaborative reflection is a form of education pertinent to many aspects of the medical curriculum [1, 2] but facilitating it can be challenging. How do you stimulate reflection? What can you, as an expert or experienced teacher, contribute to the interactional process of reflection? How can you ensure educational value when the result of group discussion depends largely on the input and dynamics of that specific group? Answers to these questions are part of teachers’ professional knowledge base, defined as “all profession-related insights, which are potentially relevant to a teacher’s activities” in a specific educational context [3]. Describing the knowledge base for facilitating collaborative reflection would be beneficial in several respects: it would improve current teacher training [4, 5], contribute to the professional development of novice teachers [3], and support conceptualizations of collaborative reflection that “take into account the complexity of real-world educational contexts” [6]. In light of these benefits, we aimed to describe the professional knowledge base for facilitating collaborative reflection by analyzing the goals and strategies formalized in documents and perceived by teachers in this context.

In general educational literature, the professional knowledge base for teaching is commonly referred to as teacher cognitions, traditionally understood as an individual teacher’s beliefs, knowledge and thoughts that drive “classroom action” [5, 7]. More recently, recognition of the influence of the social environment and professional community on these cognitions has grown [5]. Individual teacher’s cognitions often draw on discipline-based theories and concepts, pedagogical principles, and situation-specific knowledge shared by the professional community [3]. Together, this shared knowledge and a teacher’s practical knowledge of routines, procedures, and processes in actual educational situations constitute the knowledge base for teaching, which influences what teachers do in practice [8, 9].

One feature of the knowledge base for teaching that has received little attention is its interactional nature. Teaching is an interactional activity consisting mainly of talk between participants [10, 11]. This feature of the knowledge base has been described as the *professional Stocks of Interactional Knowledge*[12]: a collection of partially normative and partially descriptive ideas concerning interactional processes shared by members of the profession [13]. In the following, we will use the term *professional Stocks of Interactional Knowledge *to refer to all insights about interaction that inform teachers’ professional activities during collaborative reflection education, where an explicit focus on the interactional nature of the knowledge base is crucial.

Since there are almost no descriptions of professional Stocks of Interactional Knowledge for facilitating collaborative reflection, teachers currently do not have access to a shared resource for teaching practices. General advice on facilitating group discussion [14–16] provides a few indicators of good practice, e.g. ask thought-provoking questions, but what this entails for teachers in specific educational interactions remains unclear. In this study, we aim to bridge this knowledge gap by deconstructing normative guidelines of professional practice recorded in institutional training documents and the subjective expertise of individual teachers into a repertoire of interactional strategies that teachers can use to achieve the educational aims of collaborative reflection. Our purpose is to make the knowledge formalized in curricular documents and individual teachers’ practical knowledge available for prospective and practicing members of the professional community.

## Methods

We focus our analysis on collaborative reflection sessions at all institutes for Dutch general practitioners (GPs) in training, which provides analytic depth and specific implications for practice.

### Setting

In Dutch GP training, groups of around ten residents attend weekly sessions throughout their three-year training [17] to discuss patient cases, personal dilemmas, and other issues relevant to their training situation. One or two teachers (a practicing GP and/or a behavioral scientist or psychologist) facilitate the interactional process of collaborative reflection to generate educational value [18].

### Data and participants

We collected two types of data, covering both formalized normative guidelines and individual teacher’s practical insights on facilitating collaborative reflection. The first type of data consists of *institutional documents* from all eight Dutch GP training institutes. An overview of documents and approximate indication of their volume is presented in Tab. 1.

**Table 1:** Analyzed documents and their approximate volume in pages per institute (A–I; anonymized)

	Pages of documents included per institute								
Type of document	A	B	C	D	E	F	G	H	I
Local or national training plan	43	–	–	–	43	24	23	16	–
Information about collaborative reflection for teachers	5	19	25	1	18	12	3	3	22
Information about collaborative reflection for residents	8	–	1	–	–	7	4	6	–
Information about other reflection activities during the training (e.g. supervision)	75	20	38	2	47	35	20	41	–

The second type of data consists of 26 *video-stimulated interviews* about recorded reflection sessions with individual teachers from all eight institutes. These interviews were selected from a larger dataset of 37 video-stimulated interviews until saturation of analysis could be shown (see below). Selection was done using maximum variation sampling in terms of institute, teacher experience, and year of training. Participants in the 26 analyzed interviews were 11 GP teachers and 15 behavioral scientists with 0.5 to 18 (*M* = 7.5) years of collaborative reflection teaching experience. The recorded sessions about which the teachers were interviewed were from year 1 (7), year 2 (8) and year 3 (11) of GP training.

During the interviews, a teacher and interviewer viewed parts of a recent collaborative reflection session involving that teacher, which was video-recorded for the purposes of the study. The teacher was asked to select an interesting, difficult, smooth, or otherwise notable or memorable part of the video recording to watch together. While watching, the interviewer prompted the teacher to reflect on their actions and the underlying theoretical or practical grounds. In line with Muller [9], we view these video-stimulated reflections as in-the-moment constructions of teachers’ reportedly relevant internalized interactional norms. We describe the procedural details and grounding of this reflective approach to video-stimulated interviewing elsewhere [19]. The interviews were transcribed verbatim.

All participants consented to the video-recording of the session and the interviews. Ethical approval for this study was obtained from the Ethical Review Board of the Dutch Association of Medical Education (NVMO), dossier 829.

### Analysis

We used Template Analysis [20] to code both types of data in sequential order. Mirroring earlier work on the knowledge base for teaching in medical educational contexts [16], we decided to analyze the documents and interviews for goals (what is to be accomplished during the session) and strategies (actions that teachers can take to achieve these goals). Analytic steps and coding decisions were documented in an audit trail.

The analysis was two-phased. First, we inductively constructed an initial coding template of goals and strategies mentioned in the institutional documents. MB and MV independently coded two institutes’ documents and conferred with EG for consensus about their coding. MB and EG then independently coded two other institutes’ documents and conferred with MV for consensus. MB organized the codes into meaningful clusters of goals and strategies for facilitating collaborative reflection, discussed the resulting coding template with EG and MV, and adapted it into an initial coding template of formal, institutionalized goals and strategies for facilitating collaborative reflection.

Next, we adapted the initial template into a final template while coding goals and strategies in the video-stimulated interviews with teachers. MB coded all interviews and double-coded four interviews with EG or MV for consensus to ensure credibility. The preset criterion of saturation (no new goals and strategies in two consecutive interviews) was met after coding 26 interviews. The resulting coding template represents an organized and grounded description of goals and strategies in the data without compromising their diversity and messiness in educational practice.

## Results

The institutional documents and teacher interviews revealed a wide variety of goals for collaborative reflection sessions and a myriad of strategies to achieve them. Our collection does not represent consensus across institutes or teachers but rather the *scope* of potential goals and strategies of collaborative reflection sessions at GP training institutes, including conflicting ideas about what to achieve and how. We first present an overview of the main goals and strategies (see Table S1 of the Electronic Supplementary Material for a complete list) and then discuss a selection of these in more detail, with examples.

### Main goals

Two types of collaborative reflection goals are discussed in the documents and interviews: educational goals for residents to attain, and teacher goals to help residents attain them. Table S2 of the Electronic Supplementary Material lists the main goals in institutional documents and teacher interviews.

The main goal (from the perspective of institutes and teachers) is for residents *to learn and develop*. Learning and development is future-oriented, directed at gaining knowledge and skills to become a better doctor. While collaborative reflection sessions may seem to be “a lot of talking, not much practice,” they are actually framed by some teachers as a setting that allows residents to *work on almost all the goals of GP training* and, in doing so, to develop their professional skills, knowledge, and attitudes. This is reflected in the sub-goals, *to develop professionally, to consult peers or learn from peers*, and *to reflect or learn to reflect*. Each of these contributes in some way to the learning and development of competent GPs, who become socialized into the GP community as responsible and independent doctors. Asked her view of the main goal of collaborative reflective sessions, one teacher commented:

[The goal is] to stimulate people to reflect on their own conduct … with the eventual aim that, as a result of that reflection, they will do the things they can improve on better in future. So they will indeed become better doctors, … possibly even better people. (B851)

The role of the teacher is mostly facilitative, as reflected in the main teacher goal to facilitate the learning process. Teachers strive and are expected to strive *to have everyone participate in reflection* and *to integrate cases/stories into a theme* that is recognizable and valuable to all. These goals reflect the centrality of sharedness during collaborative reflection, not only in terms of engagement in the process, but also in the form of common experiences. Engagement in interaction is seen as positive behavior that may, some teachers argue, already be a sign of reflection. Many believe that teachers should stimulate participation and try to identify a general issue in specific cases to create educational value for all.

### Main strategies

Teachers and institutes refer to a legion of teacher strategies for attaining the resident and teacher goals described above. We summarize three main strategies.

#### (Don’t) structure and stimulate the learning process:

In our two data sources, one of the main things teachers are taught or advised to *do* (hence, a strategy) to *facilitate the learning process *is to structure and stimulate that process—or not to do so if that hampers learning and development. Sometimes facilitation involves structuring and stimulating the interactional process, for example guiding the discussion back to the main point. Other times, teachers structure and stimulate the group process at the content level, for example by checking whether the discussion is leading to the learning goals or asking useful questions that contribute to learning and development. The type of structuring and stimulation of group processes reported by teachers commonly has a didactic purpose. One example is given in the quote below, where a teacher explains how he guides his group towards “good” questions:

[The group] has to provide content and ask explorative questions, preferably ones that help … to take on a different viewpoint … . So we try to teach them … to stick to the issue [at hand], and not ask off-topic questions but ones that explore, that deepen the issue. (C806)

This quote illustrates how teachers’ strategies (in this case, teaching “good” questioning) are seen as a means to achieve a goal (in this case, to develop professional communication skills).

The relationship between teachers’ structuring and stimulation and the quality of reflective discussion is evident in almost all teacher interviews. For example, one teacher remarks that questions clarifying the issue for reflection “*facilitate in-depth discussion and help get to the resident’s learning issues faster* …” (D753). Role modeling of procedures (which provide structure, stimulate interaction) is common practice, according to some teachers, when groups have yet to familiarize themselves with collaborative reflection:

At the start, we paid a lot of attention to the procedures, so I stayed on top of things a lot and my co-teacher did too, like saying ‘No, hold on, now you’re offering a solution, you’re giving advice, so no, hold your horses.’ (E805)

Less structuring is required once residents are familiar with the procedures that stimulate reflection. Some teachers believe in sitting back and letting the residents “do the work” once the group understands the goals and how to achieve them. There is no consensus, however, on when to sit back and when to intervene. It is a balance that must be negotiated in every single reflective discussion. Ultimately, the value of whatever teachers do lies in the degree to which their actions contribute to the goal of fostering the learning and development of good future doctors.

#### Guarantee active participation of residents:

Returning to the perceived importance of participation to learning and development, we now give three examples of teacher strategies to motivate residents to participate. The first, related to the balance between structuring and letting go, is to encourage participation by *limiting one’s own contribution*. For example, if a group is seeking to solve a problem, several teachers report not (absolutely *not*) stepping in to offer a potential solution. Also, if a group falls silent, several teachers purposefully maintain the silence instead of filling it in. That may be awkward, but it can also be very valuable for learning and development: it allows room for something to “*settle in*” (B851).

The second example, linked to the first, is to actively nudge residents to talk to one another, to discuss among themselves instead of with the teacher. In one institute’s instructional documents, teachers are advised to avert their eyes when a resident is speaking as a nonverbal signal to address the other residents instead of the teacher. If that does not work, teachers are advised to ask the resident why they are addressing the teacher instead of the group. Many teachers, however, are adamant that active teacher participation is exactly what creates educational value. In their view, it is unproductive not to let residents solicit teacher contributions because the teacher is usually an expert on the topic at hand and even on how such discussions should proceed.

The third example is to actively engage residents in the discussion. This could involve encouraging a silent resident to participate, for example by telling them “*I’m missing your input*” (F897). An indirect invitation is probably more “elegant” than an explicit solicitation, according to one teacher:

[I]f I say ‘Now you have to say something,’ then I’m giving a command. Then they can be compliant or not, but then—then it suddenly becomes an issue of ‘am I going to listen to this teacher?’ But if I say to someone, for example, ‘I’m missing your input,’ ‘I haven’t heard from you yet,’ then I give them a different message. And then someone can decide for themselves like ‘oh hey, how does that feel, that apparently people would like to hear my opinion?’ (F824)

Indeed, teachers have numerous direct and indirect strategies for engaging residents in the discussion. Encouraging one resident to participate may involve hinting to others that they keep their ideas to themselves for now, or explicitly soliciting an individual’s view on the topic at hand.

Whether participation must always be active, verbal, and extensive to be perceived as supporting learning and development is an open question. One teacher’s strategy for engaging residents was to allow room for limited participation (e.g. only nonverbal), for example when something intense has just happened and spoken participation is too much. In the end, stimulating residents to participate is as much a question of monitoring what *each and every* person needs and brings as of creating *group* discussion.

#### Allow room for similar experiences:

The group nature of discussion is also reflected in the third main strategy, which teachers can use to integrate individual experiences into a common theme for discussion. Some teachers disagree about the value of such integration, as it generalizes away from specifics that may well be crucial to the problem arising in that situation. Other teachers point out the importance of identifying a common, recognizable theme to create educational value for all. One strategy mentioned in both data sources is to *allow room for similar experiences*, for example by mentioning potential themes for discussion arising from the specific experiences shared in the group, or by formulating a lesson learned at the end of a case discussion. These approaches help to identify the common thread in the issue under discussion, normalizing a potentially problematic experience while simultaneously creating opportunities for learning beyond the individual case. This, in turn, contributes to learning and development for future situations.

## Discussion

Our analysis of institutional documents and teacher interviews to identify goals and strategies used in collaborative reflection sessions at the GP training resulted in a detailed, practice-based description of the professional Stocks of Interactional Knowledge for collaborative reflection. This description serves two purposes: it informs us about the *complex structure* of that knowledge base, and it highlights the centrality of *sharedness *as a feature of interaction that can contribute enormously to the learning and development that is seen as the goal of collaborative reflection.

First, the myriad of goals and strategies reveals the dispersed nature of the knowledge base for facilitating collaborative reflection. While there is consensus about the main goals (for residents: to learn and develop; for teachers: to facilitate these processes) and shared strategies, variation prevails within that framework. This variation may clarify why so few have attempted to explicate knowledge on facilitating collaborative reflection. If we were to devise a normative guideline, we would need to survey best practices that worked in one situation and assume that these will work in similar situations, too. Given the complexity of the educational situation [6, 21], however, what to do to what end in which situation cannot be set in stone—and probably never should be if we want residents to reflect and learn [22]. The knowledge base is therefore nothing more than a contextualized overview of options showing the *scope* of collaborative reflection practice.

Second, though varied, the professional Stocks of Interactional Knowledge suggest that practices of facilitating collaborative reflection are mainly informed by an orientation on *sharedness*. Experiences are made accessible and relatable to others by extracting common themes for learning, and stimulating active participation by all residents is crucial to the reported teacher strategies. Active participation has the potential to propound multiple perspectives and diverse information, benefiting the reflective process [23, 24]. By talking about recognizable issues, residents can also become aware of their position towards full membership of the profession [24–27]. Such professional socialization [28] appears to be the ultimate goal of collaborative reflection, as revealed in our overview.

A number of considerations must be borne in mind when interpreting these conclusions. First, the overview of goals and strategies does not tell us whether strategies have the intended effects—although their origin in institutional instructions and teachers’ practice suggest perceived usefulness. Also, the goals and strategies in this synthesis are formulated in institutes’ and teachers’ own words and with reference to specific teaching situations, the latter being an artifact of the video-stimulated interviews [29]. On the one hand, this provides the necessary detail [12] and places the knowledge base firmly in practice. On the other hand, it implies that parts of the knowledge base are difficult to relate to scientific educational theory—and, therefore, have only weak substantiation in current theoretical knowledge on stimulating and engaging in collaborative reflection. That is precisely why our approach has the potential to initiate a dialogue between scientific theory and practical expertise. Additionally, integration of the perspectives of institutions and individual teachers can create an arena for connecting formal, informal, and hidden curricula [30, 31].

The multitude of goals and strategies gives novice and experienced teachers a toolkit of practices to experiment with. In our own GP training setting, we will make the knowledge base available to individual teachers as a comprehensive resource for addressing particularly challenging situations (e.g. involving passive students in the discussion). We will also use the overview for designing themed teacher training, for example about the desirability [21] of potential courses of action towards identity development. More generally, a comparison between the interviews and the institutional documents shows that the latter should be updated based on current descriptions of the knowledge base.

Moving beyond teachers’ reports, our next steps are, first, to describe residents’ perspectives on what elements of collaborative reflection create educational value, and second, to analyze recordings of collaborative reflection interaction to build a repertoire of teacher actions, and show how they are used as means to particular ends. Our description of the Stocks of Interactional Knowledge for facilitating collaborative reflection serves as a starting point for such analysis [12], as it highlights key practices and issues, and suggests how to deal with them in actual educational practice [13]. For now, our overview allows teachers to peek into one another’s “toolkit” without being physically present and while remaining anonymous—a simple way to learn the trade [32].

## Supplementary Information

Table S1. Resident aims, teacher aims, and strategies to achieve those aims during facilitation of collaborative reflection

Table S2. Overview of main goals for residents and teachers

## References

[1] Aronson L (2011). Twelve tips for teaching reflection at all levels of medical education. Med Teach.

[2] Uygur J, Stuart E, De Paor M (2019). A best evidence in medical education systematic review to determine the most effective teaching methods that develop reflection in medical students: BEME guide no. Med Teach.

[3] Verloop N, Van Driel J, Meijer P (2001). Teacher knowledge and the knowledge base of teaching. Int J Educ Res.

[4] Cantillon P, de Grave W (2012). Conceptualising GP teachers’ knowledge: a pedagogical content knowledge perspective. Educ Prim Care.

[5] Hutner TL, Markman AB (2016). Department-level representations: a new approach to the study of science teacher cognition. Sci Educ.

[6] Rotjanawongchai S, Handley Z. Capturing teacher cognition through the triangulation of interviews, observations and stimulated recall data. In: Proceedings of the Third International Conference on Language Education and Testing; 2018 Nov 26–28; Antwerp, Belgium; 2018. pp. 116–27.

[7] Fenstermacher GD (1994). Chapter 1: the knower and the known: the nature of knowledge in research on teaching. Rev Res Educ.

[8] Thiessen D (2000). A skillful start to a teaching career: a matter of developing impactful behaviors, reflective practices, or professional knowledge?. Int J Educ Res.

[9] Muller C, Pandolfi EM, Miecznikowski J, Christopher S, Kamber A (2017). Stimulated recall interview, an indicator of interactional norms internalized by language teachers. Studies on languages norms in context.

[10] Stokoe EH (2000). Constructing topicality in university students’ small-group discussion: a conversation analytic approach. Lang Educ.

[11] Gardner R (2019). Classroom interaction research: the state of the art. Res Lang Soc Interact.

[12] Peräkylä A, Vehviläinen S (2003). Conversation analysis and the professional stocks of interactional knowledge. Disc Soc.

[13] Leinonen S, Wadensjö C, Englund Dimitrova B, Nilsson A-L (2007). Professional stocks of interactional knowledge in the interpreter’s profession. The critical link 4: professionalisation of interpreting in the communicty.

[14] Azer SA (2005). Challenges facing PBL tutors: 12 tips for successful group facilitation. Med Teach.

[15] Aarnio M, Lindblom-Ylänne S, Nieminen J, Pyörälä E (2013). Dealing with conflicts on knowledge in tutorial groups. Adv Health Sci Educ.

[16] Hmelo-Silver CE, Barrows HS (2006). Goals and strategies of a problem-based learning facilitator. Interdiscip J Probl Based Learn.

[17] Veen M, de la Croix A (2017). The swamplands of reflection: using conversation analysis to reveal the architecture of group reflection sessions. Med Educ.

[18] van Braak M, Giroldi E, Huiskes M, Diemers AD, Veen M, van den Berg P (2021). A participant perspective on collaborative reflection: video-stimulated interviews show what residents value and why. Adv Health Sci Educ.

[19] van Braak M, de Groot E, Veen M, Welink L, Giroldi E (2018). Eliciting tacit knowledge: the potential of a reflective approach to video-stimulated interviewing. Perspect Med Educ.

[20] King N, Symon G, Cassell C (2012). Doing template analysis. Qualitative organizational research: core methods and current challenges.

[21] Biesta G (2007). Why “what works” won’t work: evidence-based practice and the democratic deficit in educational research. Educ Theory.

[22] Biesta G (2010). Why ‘what works’ still won’t work: from evidence-based education to value-based education. Stud Philos Educ.

[23] Mann K, Gordon J, MacLeod A (2009). Reflection and reflective practice in health professions education: a systematic review. Adv Health Sci Educ.

[24] Cruess RL, Cruess SR, Steinert Y (2018). Medicine as a community of practice: implications for medical education. Acad Med.

[25] Lave J, Wenger E (1991). Situated learning: Legitimate peripheral participation.

[26] Sfard A (1998). On two metaphors for learning and the dangers of choosing just one. Educ Res.

[27] Mann KV (2016). Reflection’s role in learning: increasing engagement and deepening participation. Perspect Med Educ.

[28] Biesta GJ, van Braak M (2020). Beyond the medical model: Thinking differently about medical education and medical education research. Teach Learn Med.

[29] Barton KC (2015). Elicitation techniques: getting people to talk about ideas they don’t usually talk about. Theory Res Soc Educ.

[30] Hafferty FW (1998). Beyond curriculum reform: confronting medicine’s hidden curriculum. Acad Med.

[31] Hafler JP, Ownby AR, Thompson BM (2011). Decoding the learning environment of medical education: a hidden curriculum perspective for faculty development. Acad Med.

[32] Boyle B, Lamprianou I, Boyle T (2005). A longitudinal study of teacher change: what makes professional development effective? Report of the second year of the study. Sch Eff Sch Improv.

