# Supplementary material for: A professional knowledge base for collaborative reflection education: a qualitative description of teacher goals and strategies
Source: Perspect Med Educ. 2021 Aug 17;11(1):53–9. doi: 10.1007/s40037-021-00677-6 (PMC8733139; doi:10.1007/s40037-021-00677-6)
Supplement: Supplementary file 1 — Table S1. Resident aims, teacher aims, and strategies to achieve those aims during facilitation of collaborative reflection [file 40037_2021_677_MOESM1_ESM.docx]

Table S1. Resident aims, teacher aims, and strategies to achieve those aims during facilitation of collaborative reflection

| **Resident aims** | **Teacher aims that contribute to resident aims** | **Strategies** |
| --- | --- | --- |
| TO LEARN AND DEVELOP | TO FACILITATE THE LEARNING PROCESS | (don't) structure and stimulate the group process - (don't) check whether process matches needs of residents  e.g. test whether people learned something from the process  e.g. ask whether group wants to discuss something further  e.g. ask what theme residents want to discuss at start of year  e.g. stimulate individual learning process  > stimulate residents to pick up a reflection issue in year 3  > ask about learning needs - monitor/set goals and results (content-wise)  e.g. check whether goals (content) are achieved  > ask 'what is your take-away? / what will you remember?'  > ask what this discussion has brought for the resident  > ask whether this discussion was enough/beneficial  > close up (evaluate, stop)  >> mention that we have to close the discussion  >> (don't) close/cut off discussion if it is not finished  >>> ask the case/story teller: 'what else would you like to tell/discuss about this?'  >> be alert to closing signals  e.g. monitor level/progression of discussion  e.g. analyze what's happening (if discussion does not run smoothly)  e.g. focus  > set boundaries  > steer discussion back to teller/concrete situation discussed  >> ask for reaction from teller on contributions from group  > ask a structuring question   >> ask for pro- and contra-arguments  > put topic/contribution on hold if it is unrelated to issue at hand  e.g. listen whether content of case/story is clear  e.g. clarify question  > check question/reason for telling case/story  > ask 'what is your aim by bringing this case/story in for discussion?'  > ask 'what is the reason that you bring this in for discussion?'  > ask 'what is your question?'  > by proposing potential issues to discuss  > search for the issue that is most 'pinching'/the hook  e.g. set the agenda  > let the group structure/determine agenda (tempo/topics)  >> let a resident chair the session  > time management  >> start with announcements  >> leave room for practical issues (if necessary)  >> social talk prior to sessions to eliminate hindering factors  > individually discuss case/story with resident if something unclear/unsolved  > alternate intense themes/stories with lighter ones (e.g. anecdote)  > postpone a case/story to the next session  > go through the agenda of the day (prior to start of the session)  > propose plan/method for discussion   > make an inventory  >> stimulate as much as needed to get adequate picture of case/story during inventory phase  >> stimulate residents to formulate question/reason for telling  >> make an inventory using themes (e.g. collaboration)  >> (don't) ask to share content of case/story in one line  >> make inventory by first discussing week in twos, then share one issue for discussion per two  >> make inventory of everyone's first patient last Monday  >> ask who wants to bring something in for discussion  >> make inventory using adjectives (surprising, emotional, irritating, fun, intense)  >> give some time to think of something to share at start of session  >> (don't) prioritize shareables  >>> (don't) prioritize using red orange green  >>> prioritize by using a b c (variation on red orange green)  >>> prioritize by asking to score shareable on importance (0-10)  >>> prioritize by urgency  >>> prioritize by having residents with low participation go first  >>> prioritize based on way of presentation (emotional?)  >>> appoint someone to be the first to share case/story  >>> don't prioritize by asking 'who wants to start?'  >>> prioritize by having the group choose order  >>>> confer with group what to discuss first if all cases/stories to tell are red (urgent)  >>> prioritize update/feedback on cases shared earlier  >>>> ensure update/feedback on cases shared earlier go first  >>> prioritize in order of people saying they have something to share  >>> prioritize in order of seating  - adaptive structuring - summarize, draw conclusions - discuss group communication (metacommunication)  e.g. ask why the group is laughing  e.g. address irritations - draw up working rules (organization, practical issues) - stimulate open communication - monitor case telling  e.g. make sure that introduction to case/story telling is short and concise  e.g. give room to tell an intense case/story  > stimulate teller of experience to continue telling  e.g. intervene when telling takes too long  > intervene by asking a question - split into subgroups and assign one teacher to each - intervene in difficult/unwanted situations  e.g. don't intervene if in doubt - keep high pace in discussion  e.g. prefer tempo over depth - stay in control - pay attention to integration of new people in the group - mark start of session - mark start of 'giving tips' (don't) use reflection tools/formats for discussion - initiate/stimulate variation in tools/ways to discuss - (don't) evaluate the used method of discussion/way discussion went/tool - situation trigger question - discuss case/story/experience just shared in twos - gossiping - something to be proud of and something  to learn about / tip and top - everyone asks 1 question, person telling story/case chooses which questions (s)he is going to answer - everyone gives a tip - do an experiment/role play (what would you say to someone in your situation?) - everyone makes a wish for someone who has just told/experienced a heavy/intense story - thematic discussion  e.g. ask which patient has stuck with you/do you still remember  e.g. ask about things that went well or which you are proud of (positive incident mentioning)  e.g. ask about learning moment that you would like to redo motivate residents to apply discussed issues in practice adapt to residents' world - take the temperature (measure how everyone 'is' today) - ask about current issues (difficult as well as easy ones)  - adapt to terminology and way of thinking used by GPs (e.g. as a psychologist) - GP teacher transforms psychologist question to GP-relevant one - make contact prior to start of session to initiate a social learning environment - slow yourself down (don't move on too quickly) improvise, adapt to what is happening - stimulate spontaneity and liveliness  e.g. strengthen what happens by hooking onto it  e.g. say 'continue asking please' to a resident asking good questions/following a useful thread  e.g. use response of other resident to stress a certain point  (don't) prepare session - post-discuss the session as teachers - discuss/align as teachers pre-session  e.g. task division between teachers  e.g. give room to other teacher to contribute in their way  > solicit participation of other teachers (give turn/task)  use theory - use the communication triangle - use paradox of change law (confirm someone’s situation, that invites their own solution/advice) - use Kolb's circle of reflection use yourself as a measure (if you feel a certain emotion, others might too) - model reactions/contributions to discussion - share one's thought process  use humor listen carefully write something down |
|  | to have everyone participate in reflection | guarantee active participation of residents - welcome a participant who comes in late and tell them what the group is at right now - limit one’s own contribution to let residents have turns  e.g. not have the first turn talking  e.g. not do much (few teacher interventions)   > dosing interventions  > by leaning back in chair  > leaving something/let something go (e.g. if you suspect it won't help the learning process) - have residents talk with each other instead of only with teachers  e.g. take different seating positions as teacher / not have a seat next to each other as teachers  e.g. make no (eye) contact with resident (to show that you are not available for input)  e.g. hand gesture to signal to resident to tell the group, not the teacher - stimulate to make use of the group  e.g. stimulate to try something out in the group - engage other residents  e.g. stimulate group to provide input (not the teacher)  e.g. ask for reactions of others  > ask whether other residents recognize something  > non-verbally invite residents to participate  >> open palm to group  >> look around  >> (don't) give a turn  > ask others 'what is the main thing you hear in this story?' - have everyone have their turn  e.g. have everyone bring something in in seating order  e.g. (don't) interrupt people who have long/many turns at talking  e.g. give less room to frequent tellers/contributors  e.g. don't force someone to contribute (telling/discussion)  e.g. give silent resident room to participate  > give turn  > solicit reaction  >> tell silent resident 'I miss you'/'I haven't heard you yet'  > stimulate silent resident to contribute more (e.g. in individual mentor conversation)  > leave room for someone to not verbally participate (e.g. if something has happened) - monitor non-verbal signals from group  e.g. look around in group - don't intervene right away when group goes silent  e.g. endorse/maintain a short silence (to let something settle in)  e.g. prevent other residents to fill up silences - stimulate to bring in cases/stories/experiences  e.g. ask 'so you haven't been at work last week?' if nothing is brought up for discussion  e.g. stimulate residents to come to session with something to discuss |
|  | to integrate cases/stories (to be) told into a theme | (don't) provide room for similar experiences  - thematize case/story told  e.g. don't mention thematic relation between two cases/stories/experiences without doing something with it  - mention potential themes for discussion during discussion - pick up on passing theme in telling, which is not in focus - formulate collaborative learning uptake at end of session |
| A. to work on almost all goals of GP training | to gain insight into relevant themes/areas of development of residents |  |
|  | - to distill themes for future education |  |
|  | - (not) to evaluate | to assess use reflection situations in assessment conversation |
|  | - to update | ask everyone to give an update of the independent practice week |
| B. to develop professionally |  |  |
| B1. to develop one's identity |  |  |
| - to learn to discuss experiences that are  important in your development as GP |  | stimulate teller of case/story to ask question to the group that stays close to self |
| * to discuss and learn to deal with mistakes |  | make mistakes discussable  e.g. label mistakes positively  e.g. invite group to collaboratively learn from mistakes |
| * to discuss successes |  | ask about successes/fun anecdotes stress that positive experiences may also be brought up for discussion |
| * to discuss issues concerning training situation  (GP trainer, learning path) |  |  |
| - to become part of the profession |  |  |
| * to explore the world of the GP(-in-training) |  | stimulate to explore task description of GP |
| * to learn to recognize own identity within  professional identity |  |  |
| # to learn to take more freedom to make own  choices |  |  |
| # to form own opinion |  |  |
| - to get to know oneself |  |  |
| B2. to develop professional skills | to show residents expert examples of professional behavior | be a role model  e.g. (don't) bring in own experience  e.g. use duo teachership as tool |
| - to develop communication skills |  |  |
| * to develop advice-giving skills |  |  |
| * to train to deal with conflict |  |  |
| * to practice consultation skills (e.g. question  clarification) |  |  |
| * to develop feedback skills |  | stimulate feedback giving between residents according to feedback rules |
| * to learn to ask difficult/critical/emotional  questions |  | ratify questions/interaction between residents  e.g. come back to/address valuable contribution that was not picked up in discussion stimulate listening attitude   e.g. stimulate residents to not foreground themselves in contributions that are to help others   by asking 'what is your question to the teller resident?'  e.g. cut off judgments  e.g. make sure advice is not given too early  e.g. put a question/response of another resident on hold  e.g. provide a nuance / transform a contribution (e.g. from statement to question) stimulate to ask questions about core topic of discussion stimulate to ask open questions |
| * to learn to chair |  |  |
| - to develop social skills |  |  |
| - to learn to be open to other perspectives and  understand these |  |  |
| - to develop assertiveness | - to promote autonomy | stress agency of residents   e.g. "you can CHOOSE, you don't HAVE to do something" |
| C. to consult peers/peer learning |  |  |
| C1. (not) to get answers/advice/solutions | - give residents (content/process) feedback | ask for clarification of the advice given by residents correct (e.g. miscommunication) nod give a compliment encourage weak participants, slow down over non-confident participants use reactions of group to address emotion  e.g. mention something you notice (e.g. agitation) formulate feedback as general feedback, not addressed at one individual give feedback by formulating it as your own feedback as teacher |
| - to learn to provide content in discussion |  | give non-teller resident feedback on their contribution (e.g. question, advice) |
| - to learn to use peers to find a solution | - to let residents find a solution on their own | provide room for exceptional medical cases use expertise of residents return question to the group return question to the one asking the question stimulate residents to ask for clarification of questions by other residents encourage resident to say what they themselves want (don't) provide content (solutions, tips, advice)  e.g. (don't) bring in own expertise  e.g. (don't) stress an important insight/advice/solution/etc. by resident  e.g. consider whether a teacher contribution is functional (if only interesting for teacher, then don't intervene)  > don't engage with blind spots and favorite topics of teacher  e.g. (don't) interpret  e.g. (don't) have residents look something up and get back to it next time  e.g. bring in another perspective |
| * to learn from others' practice experiences by  exchanging experiences |  |  |
| - to formulate an action plan |  |  |
| C2. to search for/provide (emotional) support | - to provide guidance / a hold to survive / cope in internship | make emotional connection provide room for feelings  e.g. notice and ask about emotionally laden words  e.g. ask about feelings  e.g. give emotional reflections  e.g. ask whether discussion of a case/story actually has more priority than resident indicates  e.g. pay attention to (visible) emotions support, help |
| - to search for/provide  recognition/acknowledgement/understanding |  |  |
| C3. to provide a frame of reference / to normalize |  | (don't) comfort resident give confirmation (from GP experience, expert opinion) test against norm |
| - to normalize bizarre/complex/difficult  situations (e.g. unexpected death) |  |  |
| - to gain confidence in own competences |  | enhance resident confidence (in own competence/conduct) "strengthen" resident by encouraging, positive evaluation |
| C4. "to meet" |  |  |
| D. to (learn to) reflect |  | (don't) explicitly discuss/let residents experience importance/value of reflection stimulate reflection |
| D1. to free space to for new learning experiences |  |  |
| - to vent steam/to spout |  |  |
| - to share wonder |  |  |
| - not to just tell an experience/babble along |  |  |
| D2. to mention and use points for learning | - to recognize patterns in stories told/reactions |  |
| D3. to discuss learning goals of supervision/  connect experiences to supervision |  |  |
| D4. to give meaning to conduct or situations |  |  |
| D5. to learn to connect subjective experiences to  others' |  |  |
| D6. to become aware of one's own conduct | - to 'hold up a mirror' | stimulate awareness of own conduct/attitude  e.g. pause discussion, slow down |
| D7. to reflect on actual situation (no abstract  discussion) |  |  |
| - to gain insight in a situation |  | stimulate to make situation more concrete  e.g. explore  > ask questions  >> ask open questions  >> (don't) ask questions with suggestions for answers (own ideas)  >> (don't) question/dig deeper  e.g. ask for clarification   e.g. repeat a word to invite someone to elaborate  e.g. concretize by asking for meaning of specific word that someone uses   > " 'holding back', what does that mean for you?"  > address uses of "to try" by asking "did you do it? if not, why not? if yes, why did you fail?"  e.g. steer and ratify explorations |
| * to discuss how you feel, think, act in a  situation |  |  |
| * to increase insight into  thoughts/feelings/conduct of patient |  |  |
| * to gain insight in communication between  you and patient |  | stimulate to take a different perspective bring various perspectives to the fore |
| * to discuss different perspectives on the  situation |  | deepen the discussion |
| D8. to deepen experiences |  | connect theory to an experience |
| D9. to connect practice experiences and theory |  |  |
| D10. to learn to question oneself |  |  |
| D11. to present one's vulnerability in a safe learning climate | - to create safety | introduction to each other at start of the group give room to residents to indicate own limits (during discussion)  e.g. ask resident whether they want to discuss something in the group (invite to) come back to something in the next session check how a response is received by a resident create open/informal atmosphere  e.g. make non-verbal contact with residents  > position teacher chair such that teacher is directed toward resident  e.g. prepare tea do not judge, respect each other's contribution/opinion  e.g. discuss breaching of rules concerning safety, respect, etc. |
