# Supplementary material for: A professional knowledge base for collaborative reflection education: a qualitative description of teacher goals and strategies
Source: Perspect Med Educ. 2021 Aug 17;11(1):53–9. doi: 10.1007/s40037-021-00677-6 (PMC8733139; doi:10.1007/s40037-021-00677-6)
Supplement: Supplementary file 2 — Table S2. Overview of main goals for residents and teachers [file 40037_2021_677_MOESM2_ESM.docx]

Table S2. Overview of main goals for residents and teachers

| **Educational goals for residents** | **Teacher goals that help residents attain goals** |
| --- | --- |
| TO LEARN AND DEVELOP | TO FACILITATE THE LEARNING PROCESS  to have everyone participate in reflection  to integrate cases/stories into a theme |
| A. to work on almost all goals of GP training | to gain insight into relevant themes/areas of development of residents |
| B. to develop professionally |  |
| B1. to develop one's identity |  |
| B2. to develop professional skills | to show residents expert examples of professional behavior |
| C. to consult peers/peer learning | give residents content/process feedback |
| C1. (not) to get answers/advice/solutions |  |
| C2. to search for/provide (emotional) support | to provide guidance/a foothold to survive/cope with the situation |
| C3. to provide a frame of reference/to normalize |  |
| C4. "to meet" |  |
| D. to (learn to) reflect |  |
| D1. to free up space for new learning experiences |  |
| D2. to mention and use points for learning | to recognize patterns in stories told/reactions to stories |
| D3. to discuss learning goals of supervision/  connect experiences to supervision |  |
| D4. to give meaning to conduct or situations |  |
| D5. to learn to connect subjective experiences to those of others |  |
| D6. to become aware of one's own conduct | to “hold up a mirror” |
| D7. to reflect on actual situations (no abstract  discussion) |  |
| D8. to deepen experiences |  |
| D9. to connect practice experiences and theory |  |
| D10. to learn to question oneself |  |
| D11. to present one’s vulnerability in a safe  learning climate | to create safety |
